# Aerosolized Bovine Lactoferrin Counteracts Infection, Inflammation and Iron Dysbalance in A Cystic Fibrosis Mouse Model of *Pseudomonas aeruginosa* Chronic Lung Infection

**DOI:** 10.3390/ijms20092128

**Published:** 2019-04-30

**Authors:** Antimo Cutone, Maria Stefania Lepanto, Luigi Rosa, Mellani Jinnett Scotti, Alice Rossi, Serena Ranucci, Ida De Fino, Alessandra Bragonzi, Piera Valenti, Giovanni Musci, Francesca Berlutti

**Affiliations:** 1 Department of Biosciences and Territory, University of Molise, 86090 Pesche, Italy; giovanni.musci@unimol.it; 2 Department of Public Health and Infectious Diseases, University of Rome La Sapienza, 00185 Rome, Italy; mariastefania.lepanto@uniroma1.it (M.S.L.); luigi.rosa@uniroma1.it (L.R.); mellanijinnett.scotti@uniroma1.it (M.J.S.); piera.valenti@uniroma1.it (P.V.); francesca.berlutti@uniroma1.it (F.B.); 3 Infections and Cystic Fibrosis Unit, Division of Immunology, Transplantation and Infectious Diseases, IRCCS San Raffaele Scientific Institute, 20132 Milano, Italy; rossi1.alice@hsr.it (A.R.); ranucci.serena@hsr.it (S.R.); defino.ida@hsr.it (I.D.F.); bragonzi.alessandra@hsr.it (A.B.)

**Keywords:** cystic fibrosis, bovine lactoferrin, iron, ferroportin, lung infection, inflammation

## Abstract

Cystic fibrosis (CF) is a genetic disorder affecting several organs including airways. Bacterial infection, inflammation and iron dysbalance play a major role in the chronicity and severity of the lung pathology. The aim of this study was to investigate the effect of lactoferrin (Lf), a multifunctional iron-chelating glycoprotein of innate immunity, in a CF murine model of *Pseudomonas aeruginosa* chronic lung infection. To induce chronic lung infection, C57BL/6 mice, either cystic fibrosis transmembrane conductance regulator (CFTR)-deficient (Cftr^tm1UNC^TgN(FABPCFTR)#Jaw) or wild-type (WT), were intra-tracheally inoculated with multidrug-resistant MDR-RP73 *P. aeruginosa* embedded in agar beads. Treatments with aerosolized bovine Lf (bLf) or saline were started five minutes after infection and repeated daily for six days. Our results demonstrated that aerosolized bLf was effective in significantly reducing both pulmonary bacterial load and infiltrated leukocytes in infected CF mice. Furthermore, for the first time, we showed that bLf reduced pulmonary iron overload, in both WT and CF mice. In particular, at molecular level, a significant decrease of both the iron exporter ferroportin and iron storage ferritin, as well as luminal iron content was observed. Overall, bLf acts as a potent multi-targeting agent able to break the vicious cycle induced by *P. aeruginosa*, inflammation and iron dysbalance, thus mitigating the severity of CF-related pathology and sequelae.

## 1. Introduction

Cystic fibrosis (CF) is a genetic disorder affecting several organs and reducing expectancy and quality of life. The most relevant damages are observed in the airways that are inherently prone to infection. Airway infections begin in early life [1] and by adulthood, they became chronic. Even though the microbial epidemiology has changed in the last years, it is well established that the chronic airway infections are often sustained by *Pseudomonas aeruginosa* [2,3]. During CF-induced airway infection progress, *P. aeruginosa* gradually shifts from the virulent pathogen form of early infection to the host-adapted pathogen form typical of the chronic phase, characterized by biofilm lifestyle and antibiotic resistance [4]. *P. aeruginosa* chronic infection correlates to increased rate of lung function decline, morbidity, and mortality [5].

Airways inflammation is a hallmark of CF, being present even before bacterial colonization and detectable infection, as demonstrated by high levels of interleukin (IL)-8 and accumulation of neutrophils in bronchoalveolar lavage fluid (BALF), and in general to activation of numerous pro-inflammatory genes dependent from nuclear factor kappa-light-chain-enhancer of activated B cells (NF-κB) and activator protein (AP)-1 pathways [6,7,8]. Inflammation is related to the prolonged inflammatory response in the lung that persists even after the inflammatory stimulus is over [9]. As a matter of fact, the massive recruitment of leukocytes into the lung airways gives rise to a self-enhancing loop, where the infiltrated neutrophils undergo necrosis and release proteases and chemoattractant molecules, thus leading to tissue damage and to the further recruitment of leukocytes. Furthermore, neutrophils are highly activated because of the genetic defect thus increasing lung damage [10].

In addition to infection and inflammation, a dysbalance of iron homeostasis is observed in CF airways and high levels of iron (up to >100 μM) in airway secretions can be recorded [11,12,13]. Iron loss into the sputum negatively correlates with cell lung functionality, while it positively correlates with cell and tissue damage and disease severity [11,14]. Iron excess can derive from the altered expression of the main proteins involved in iron homeostasis. In particular, an increased expression of both ferroportin (Fpn), the sole mammalian iron exporter [15], and ferritin (Ftn), the main iron storage protein, in lung tissue of CF patients has been observed [16]. Moreover, elevated concentrations of both Ftn and transferrin (Tf), the main iron transport protein in blood, in the lung lavage of CF patients were found [16]. Overall, all the changes in Fpn, Ftn and Tf expression converge in high rates of iron accumulation in the lower respiratory tract of CF subjects [13].

The iron overload in CF airways favours the growth and the biofilm lifestyle of *P. aeruginosa*, thus worsening the inflammatory status and host damage [4,12,17,18,19,20]. Bacterial biofilm invades CF airway epithelial cells that respond by activating a strong inflammatory response [21,22]. Therefore, infection, inflammation and iron disorders reinforce each other, establishing an unsafe vicious circle difficult to counteract and solve in vivo.

Lactoferrin (Lf), a cationic glycoprotein able to chelate two Fe^3+^ ions per molecule with high affinity, is synthesized by exocrine glands and neutrophils in infection and inflammation sites [23]. Lf is a component of natural immunity exerting multiple functions both dependent and independent of its iron-withholding ability and represents a major endogenous anti-microbial constituent of airway secretions [23,24]. The milk derivative bovine Lf (bLf) shares high sequence homology with the human protein, showing identical properties [23]. In particular, unrelated to its iron-withholding function, bLf inhibits the host cell invasion by some intracellular facultative or obligate bacterial pathogens [22,23,25,26,27]. Noteworthy, it exerts a potent anti-inflammatory activity, contributing to mucosal protection from inflammation-related damage [21,22,24,25,28,29,30,31,32].

Interestingly, our group recently demonstrated that bLf is involved in the modulation of iron homeostasis [33]. In different cell models, either infected or lipopolysaccharide (LPS)-stimulated, bLf was found to be able to modulate the synthesis of Fpn, Ftn, transferrin receptor (TfR)-1, and membrane-bound ceruloplasmin [22,34,35]. Finally, we recently investigated the role of aerosolized bLf against infection and inflammation in murine models of acute and chronic *P. aeruginosa* lung infection [36]. Interestingly, we showed that bLf was efficient in reducing, yet not significantly, bacterial load in both acute and chronic infection, compared to the control groups. Regarding inflammation, bLf-treated mice showed a significant decrease of several pro-inflammatory cytokines as well as of total leukocytes and neutrophils counts in BALFs for both acute and chronic infection models, compared to saline treated ones [36].

Here, we explore the efficacy of aerosolized bLf in counteracting infection and inflammation as well as iron dysbalance in a CF mouse model of chronic *P. aeruginosa* lung infection. Notably, the C57Bl/6 strain of wild-type (WT) littermates used as controls in this study and derived from Jackson was different from that analysed in the previous study from Charles River [36].

## 2. Results

### 2.1. Effect of Aerosolized bLf on Body Weight in Mice Infected by P. aeruginosa

To mimic a chronic lung infection similar to the one typically established in the lungs of CF patients, mice were inoculated by intratracheal (i.t.) injection with *P. aeruginosa* multidrug-resistant MDR-RP73 strain embedded in agar beads [37]. Notably, the chronic infection model obtained by inoculating bacterial cells in an immobilizing agent, such as agar, favours long-term bacteria persistence in the lungs causing airway inflammation and damage. This model differs from acute pneumonia where administration of planktonic bacterial cells in mice is rapidly cleared by the host. In this work, the *P. aeruginosa* chronic infection was carried out for seven days. Although in the early phase of chronic infection, in our experience the bacterial load and percentage of infected mice from seven days to one month after infection are similar, indicating that this model develops a stable chronic infection [37].

Local treatment with 200 µg of bLf or sterile saline by a MicroSprayer Aerosolizer device (Penn-Century Inc., Windmoor, PA, USA) started five minutes after infection and was repeated daily for a total of seven administrations.

**Figure 1:**
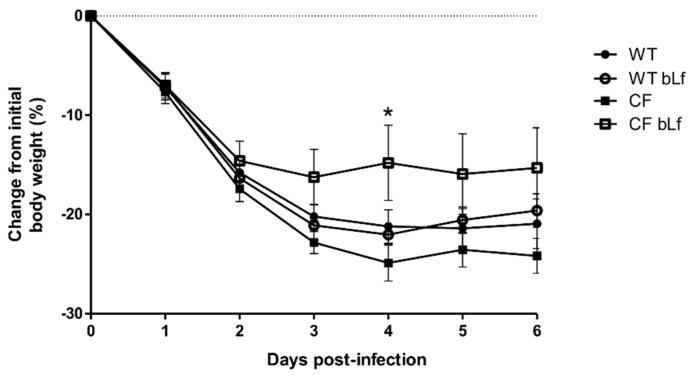
Changes in body weight of wild-type (WT) and cystic fibrosis (CF) mice suffering from chronic lung infection with *Pseudomonas aeruginosa* MDR-RP73 over 6 days (7 treatments with aerosol administration of 50 µL sterile saline or 200 µg/50 µL bovine lactoferrin (bLf)). Statistical significance is indicated as follows: *: *p* < 0.05 (Two-way ANOVA with Bonferroni’s multiple comparison test).

The body weight is considered as an indicator of well-being [38]. Therefore, the impact of bLf treatment on this read-out was recorded. The body weight of the infected mice decreased rapidly after infection for all groups (Figure 1). After six days of chronic infection and seven treatments, bLf-treated CF mice had significantly lower loss of body weight compared to the group of vehicles. No difference between bLf-treated WT mice and vehicle was observed with poor recovery in body weight (Figure 1).

### 2.2. Effect of Aerosolized bLf on Bacterial Growth in Murine Lung Infected by P. aeruginosa

**Figure 2:**
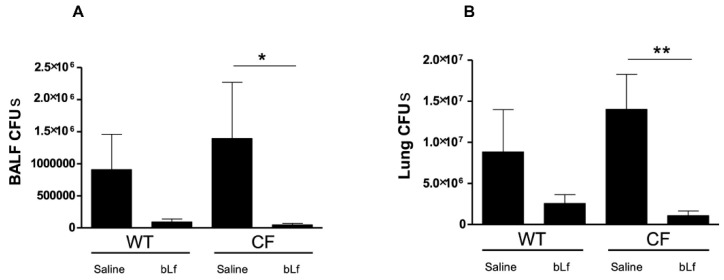
*P. aeruginosa* counts in bronchoalveolar lavage fluid (BALF) (**A**) and lung homogenates (**B**) of WT and CF mice after 7 treatments with aerosol administration of 50 µL sterile saline or 200 µg/50 µL bLf (see Materials and Methods section for details). Error bars: standard error of the mean. Statistical significance is indicated as follows: *: *p* < 0.05; **: *p* < 0.01 (Mann Whitney U test).

To evaluate the antibacterial effect of aerosolized bLf, *P. aeruginosa* counts were performed in both lung homogenates and BALFs (Figure 2). A reduction of *P. aeruginosa* Colony Forming Units (CFUs) in bLf-treated vs. untreated WT mice was observed both in BALFs and lung homogenates but differences did not reach statistical significance. On the other hand, a significant reduction of *P. aeruginosa* counts was recorded in both BALFs and lung homogenates of bLf-treated CF mice compared with untreated controls (Figure 2A,B).

### 2.3. Effect of Aerosolized bLf on Inflammatory Response in Murine Lung Infected by P. aeruginosa

**Figure 3:**
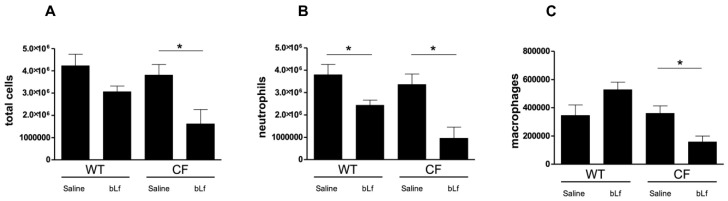
Total cell (**A**), neutrophils (**B**) and macrophages (**C**) counts in BALFs of WT and CF mice after 7 treatments with aerosol administration of 50 µL sterile saline or 200 µg/50 µL bLf, evaluated 6 days after challenge (see Materials and Methods section for details). Error bars: standard error of the mean. Statistical significance is indicated as follows: *: *p* < 0.05 (Mann-Whitney U test).

Next, we evaluated the effect of bLf on host immune cells recruitment in the BALF and cytokine/chemokine production in lung tissues of WT and CF mice following *P. aeruginosa* infection. As shown in Figure 3, significant (*p* < 0.05) reductions of neutrophils, macrophages and total cells were recorded for CF mice, while only a significant reduction in neutrophils was observed for WT mice treated with bLf compared with saline-treated controls.

**Figure 4:**
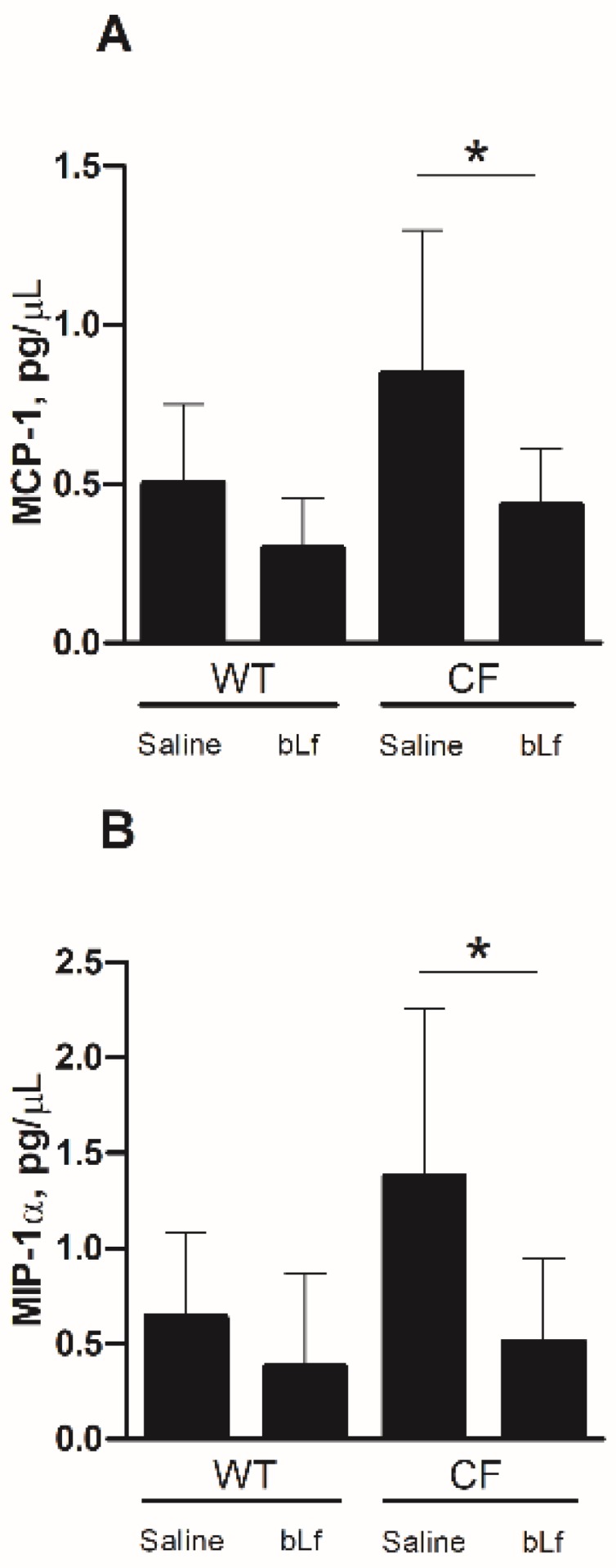
Changes in Monocyte Chemoattractant Protein (MCP)-1 and Macrophage Inflammatory Protein (MIP)-1α levels in lung homogenates of WT and CF mice after 7 treatments with aerosol administration of 50 µL sterile saline or 200 µg/50 µL bLf. Error bars: standard error of the mean. Statistical significance is indicated as follows: *: *p* < 0.05 (Mann-Whitney U test)**.**

Cytokine/chemokine profiles showed that IL-2, IL-4, IL-9 and granulocyte-macrophage colony-stimulating factor (GM-CSF) were not expressed at all or were expressed at very low levels. Out of 18 cytokine/chemokines detected, the most striking result was obtained with Monocyte Chemoattractant Protein (MCP)-1 and Macrophage Inflammatory Protein (MIP)-1α. As shown in Figure 4, levels of these chemokines were higher in transgenic CF mice compared to WT mice and bLf-treatment significantly reduced their concentrations to levels of untreated WT mice. Other cytokine/chemokine showed higher levels in CF compared to WT mice and a trend in reduction, yet not significant, for bLf-treatment. Table 1 summarizes analytical data for all cytokines/chemokines tested.

### 2.4. Effect of Aerosolized bLf on Proteins of the Iron Homeostasis System of Mice Infected by P. aeruginosa

**Figure 5:**
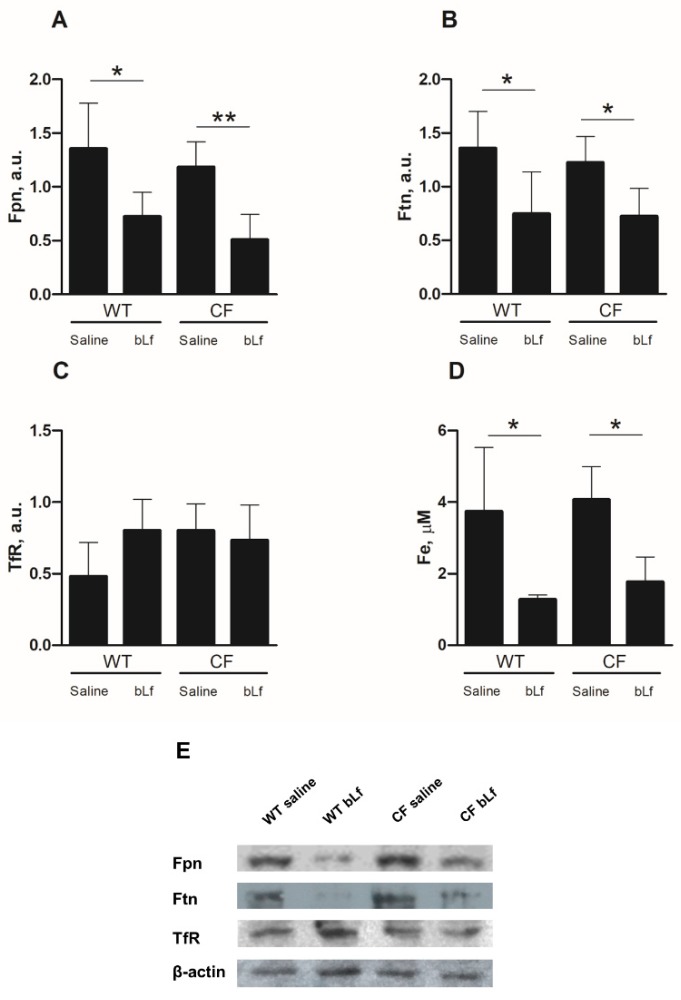
Changes in ferroportin (**A**), ferritin (**B**), transferrin receptor (**C**) levels in lung homogenates analyzed by Western blot and total iron content (**D**) in BALFs of WT and CF mice after 7 treatments with aerosol administration of 50 µL sterile saline or 200 µg/50 µL bLf. (**E**) Representative Western blot. Error bars: standard error of the mean. Statistical significance is indicated as follows: *: *p* < 0.05; **: *p* < 0.01 (Mann-Whitney U test). a.u: arbitrary unit.

To investigate the role of bLf on iron homeostasis of mice infected by *P. aeruginosa*, lung homogenates of WT and CF mice, treated with saline or bLf, were analysed for the expression of the main iron-related proteins involved in cell and systemic iron homeostasis, namely Fpn, Ftn and TfR1, and total iron content in the BALF was also assessed. As shown in Figure 5A,B, a reduced expression of Fpn and Ftn both in WT and CF infected mice was observed upon treatment with bLf. For both proteins, reduction was statistically significant both for WT and CF animals (*p* < 0.05). On the other hand, TfR1 was unaffected by bLf administration (Figure 5C). As far as total iron content in BALF is concerned, a significant decrease in bLf treated mice, both WT and CF, could be observed, with a mean drop of 65% and 55% in the case of WT and CF animals, respectively (Figure 5D).

## 3. Discussion

Despite the improvement of infection therapy based on aerosolized antibiotics [39], chronic *P. aeruginosa* infections remain very difficult to treat [40,41]. Aggressive antibiotic and anti-inflammatory treatments may ameliorate CF patient symptoms in the short-term, but they do not consistently reduce inflammation and bacterial load, which may be related to persistently elevated iron concentrations in the airway [13]. So far, it has not been established whether iron dyshomeostasis in lung is a cause or a consequence of *P. aeruginosa* infection in CF, while it is known that decreasing available iron generally ameliorates lung injury associated to inflammation states [42,43]. Therefore, since inflammation is a hallmark also in CF, iron is likely to be a major actor in the maintenance of this disease.

In CF airways, iron dysbalance is mainly correlated to an increased expression of two pivotal proteins of iron homeostasis, namely Ftn and Fpn [16]. With the present study, we shed some light on the role of bLf treatment on relieving iron dysbalance in a CF mouse model of *P. aeruginosa* chronic lung infection. The use of bLf is justified by the fact that bLf is generally recognized as a safe substance by Food and Drug Administration (USA) and available in large quantities. Therefore, bLf is utilized in the majority of the in vitro studies as well as in clinical trials to identify putative applications [44,45,46,47,48]. Our results are consistent with previous findings from our group demonstrating that aerosolized bLf was efficient in diminishing both neutrophil recruitment and pro-inflammatory cytokine levels in pre-clinical mouse models of *P. aeruginosa* acute and chronic lung infection [36].

Here, we show for the first time that bLf treatment is significantly efficient in reducing bacterial load in both BALFs and lung homogenates in CF mice, compared with saline-treated ones. As far as the WT controls are concerned, the results confirm our previous data that no significant difference in CFUs counts between bLf and saline-treated mice has been observed [36]. The central role of bLf as an anti-inflammatory agent is confirmed by the significant decrease of both total immune cells, such as neutrophils and macrophages, and some pro-inflammatory cytokines in CF compared to WT mice. In particular, MCP-1 and MIP-1α levels were significantly reduced in bLf-treated CF mice with respect to controls. It is interesting to note that these cytokines are involved in the migration of monocytes/macrophages [49,50] and that the migration of blood into the airway supports neutrophil-mediated tissue injury in CF [51]. Thus, the observed decrease of both MCP-1 and MIP-1α levels suggests a reduced pro-inflammatory monocytes infiltration of CF mouse airway upon bLf treatment.

In association with the anti-bacterial and anti-inflammatory activity of bLf, we demonstrate, for the first time, the ability of bLf to counteract lung iron disorders in both WT and CF mice infected by *P. aeruginosa*, by reducing iron overload, along with a drop of detoxifying proteins in lung homogenates and of iron concentration in BALF. In particular, bLf-treated mice, both WT and CF, showed a marked reduction in both Fpn and Ftn expression when compared to saline-treated ones. Ferroportin and ferritin are considered hallmarks of iron overload in lung epithelium, that reacts to excess iron by upregulating proteins able to trap (Ftn) or export (Fpn) the metal into the lumen. It is interesting to note that Fpn is usually downregulated in conditions of iron overload [52] since its primary localization is on the basal membrane of enterocytes and on macrophages, both cells being in charge of refurnishing circulating blood with iron through Fpn when needed. In our experimental set, the high expression levels observed for both proteins in both WT and CF infected animals are consistent with a dysregulation of lung iron homeostasis that is presumably induced in WT mice, and exacerbated in CF ones, by *P. aeruginosa* infection. The effect of bLf is striking in both cases, with a marked reduction of the proteins’ levels. Since iron levels in BALFs are also decreased in bLf-treated mice, our results allow to hypothesize that treatment with bLf, along with its anti-bacterial activity, may also prevent iron entry into the epithelial layer, thus inducing a decrease in intracellular iron which in turn downregulates Ftn and Fpn and makes iron concentrations in the airway lumen secretion drop. Certainly, more experiments are required to demonstrate and confirm this putative mechanistic model. However, this effect, which is related to a model of chronic infection, is different from what we have observed in the case of acute infection of CF polarized epithelium with the *P. aeruginosa* strain LESB82, where Fpn synthesis is upregulated [22]. Concerning TfR1 expression, no significant difference between saline- and bLf-treated mice was observed. Notably, Heilig et al. [53] reported that TfR1 levels in whole lung remained unchanged in condition of iron overload, while an increase in Ftn levels was observed. However, since TfR1 has been described to be localized on both basolateral and apical side of airways epithelium [52], a dysregulation of its local expression cannot be ruled out.

Recently, the bLf ability in modulating iron homeostasis disorders has been reported in in vitro models of infected human CF and intestinal epithelium as well as in LPS-challenged human monocytes/macrophages [22,34,35]. This modulation has been demonstrated to be correlated to the concomitant down-regulation of IL-6, the main pro-inflammatory cytokine involved in iron homeostasis [54,55,56]. However, at odds from this Fpn positive modulation exerted by bLf in epithelial and macrophagic models, here we show that bLf decreases pulmonary Fpn expression in both WT and CF mice, with no modulation of either IL-6 or other pro- or anti-inflammatory cytokines directly involved in iron homeostasis. This intriguing result highlights, once again, how bLf exerts its multifunctional activities depending on the cell system it acts upon [22,23,30,57]. In this respect, we have to recall that pulmonary Fpn, as well as Ftn, seems to be related to an iron detoxifying function [52], rather than to systemic iron transport and recycling, activities which are instead associated to the main cells involved in iron homeostasis, namely enterocytes, macrophages, and hepatocytes.

**Figure 6:**
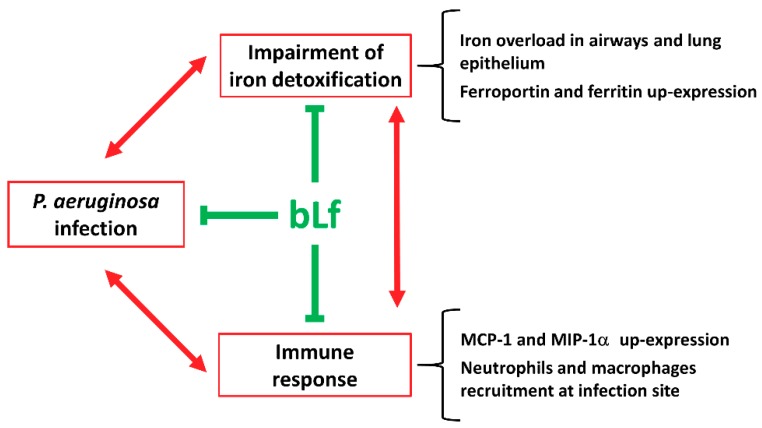
Summary diagram on bLf multi-targeting activities (green T bars) counteracting the unsafe vicious cycle established by infection, inflammation and iron dysbalance (red arrows) in CF mice suffering from *Pseudomonas aeruginosa* chronic lung infection.

The finding of diminished iron concentrations in BALFs of bLf-treated mice, both WT and CF, is of outmost importance because of the biological relevance of excessive iron in enhancing bacterial growth and pathogenesis as well as chronic inflammatory states. Overall, these results allow us to assert that bLf, along with its anti-bacterial and anti-inflammatory functions, can act as a clearing agent, able to restore the physiological iron detoxification pathway, thus leading to a progressive decrease of iron concentrations in both airways and lung epithelium (Figure 6). In this respect, the decrease of both Fpn and Ftn, after seven days of bLf treatments, can be considered as a signal of an efficient iron clearance in lung epithelium, with the cells no longer needing to detox intracellular excess iron by Ftn storage and Fpn-mediated export. To our knowledge, lactoferrin is the first molecule described as able to act, directly or indirectly, against infection, inflammation and iron dysbalance (Figure 6).

These findings add new insights into the potential therapeutic applications of aerosolized lactoferrin. Overall, bLf acts as potent multi-targeting agent able to break the vicious cycle induced by pathogens, inflammation and iron disorders, thus mitigating the severity of CF-related pathology and sequelae.

## 4. Materials and Methods

### 4.1. Bacterial Strain, Media, and Culture Conditions

MDR-RP73, a *P. aeruginosa* clinical strain isolated from a CF patient suffering from chronic lung infection [58], was tested for purity on Trypticase Soy Agar (TSA) plates (Becton Dickinson, Sparks, MD, USA) and grown to exponential phase in Tryptic Soy Broth (TSB) (Becton Dickinson, Sparks, MD, USA). Prior infection, bacteria were pelleted (2700× *g*, 15 min, 4 °C), washed twice with sterile phosphate buffer saline (PBS) and embedded in agar-beads as previously described [37].

### 4.2. Lactoferrin

Bovine milk derivative Lf (bLf), at high purity and integrity rates, was generously provided by Morinaga Milk Industries Co., Ltd. (Tokyo, Japan). BLf iron saturation was of 15%, while LPS contamination, checked by Limulus amebocyte assay (LAL Pyrochrome kit, PBI International, Italy), was found to be 0.55 ± 0.04 ng/mL [59]. Before use, bLf saline solutions were sterilized through 0.2 µm filters (Millipore Corp., Bedford, MA, USA).

### 4.3. Animals, Chronic Infection and Treatments

Animals employed in all procedures were 11–14 week-old gut-corrected CFTR-deficient C57BL/6 Cftr^tm1UNC^TgN(FABPCFTR)#Jaw mice (CF mice) and their WT littermates. Mice were originally obtained from Case Western Reserve University and maintained at San Raffaele Scientific Institute (Milan, Italy). Throughout all experimental procedures, in order to maintain definite pathogen-free conditions, mice were retained in sterile ventilated cages. Fluorescent lights were cycled 12 h on, 12 h off, and ambient temperature (23 ± 1 °C) and relative humidity (40–60%) were controlled. Mice were fed with irradiated 5K-52 rodent chow (Safe, Augy, France) and autoclaved tap water. Intraperitoneal injection of 2.5 mg of 2,2,2-tribromethanol (Avertin, Sigma-Aldrich, St. Louis, MO, USA) in saline solution was administered (15 µL/g volume/body weight) to anesthetize mice. A ventral midline incision was carried out to directly visualize and expose the trachea. Mice were intubated with a sterile and flexible 22-gauge catheter (Becton Dickinson, Madrid, Spain) attached to a 1 mL syringe [37]. To establish the chronic infection, mouse lungs were inoculated with 50 µL of an agar bead suspension containing 1.0 ± 0.1 × 10^6^ CFUs of *P*. *aeruginosa* MDR-RP73 to mimic biofilm lifestyle [37,60,61]. *P. aeruginosa* strain was implanted via the cannula into the lung with both lobes inoculated.

36 CF and 31 WT mice were randomly assigned to the bLf or saline group. Aerosolized treatments with saline (control group) or bLf (200 µg/50 µL of freshly prepared solution) were performed by using the micro-spray aerosol device (MicroSprayer aerosolizer model IA-C and FMJ-250 high-pressure syringe; Penn-Century Inc., Windmoor, PA, USA). Administrations were performed 5 min after infection under anaesthesia (5% isoflurane–oxygen, running at 4 L/min) according to established procedures. BLf or saline treatments were repeated daily for seven days. As previously reported [37,62], mice suffered inoculation of agar beads and related procedures with major differences between WT and CF mice. In particular, 42% of CF mice (15 CF) and 80% of WT (25 WT) survived the infection. Thus, the final groups were composed by: 8 CF-vehicle treated, 7 CF-bLf treated and 11 WT-vehicle treated, 14 WT-bLf treated. All mice survived over seven days treatment and were sacrificed six hours after the last treatment.

### 4.4. Broncho-alveolar Lavage (BAL) Fluid Collection and Analysis

A total of 1 mL of RPMI 1640 with protease inhibitors (Complete tablets, Roche Diagnostic, Basel, Switzerland) was used to perform the bronchoalveolar lavage (BAL) through a 22-gauge venous catheter. The lavages were repeated three times. The obtained BALFs were aliquoted and serially diluted 1:10 in PBS. Aliquots were used to count *P. aeruginosa* CFUs and total cells. To perform neutrophils and macrophages counts, BALF aliquots were centrifuged (330× *g* for 8 min at 4 °C). The supernatants were collected and stored at −80 °C and the resulting pellets were resuspended in RPMI, containing 10% fetal bovine serum (FBS), in order to obtain a final cell suspension of about 1 × 10^6^ cells/mL. A total of 150 µL of cell suspension was used to determine differential cell count by cytospins stained with Diff Quick (Medion Diagnostic, Düdingen, Switzerland).

### 4.5. Lung Homogenates

Aseptically removed lungs were homogenized in 2 mL PBS with protease inhibitors. PBS serially 1:10 dilutions of lung homogenates were performed to count *P. aeruginosa* as above described. Afterward, lung homogenates were pelleted by centrifugation (16,000× *g* for 30 min at 4 °C) and the resulting supernatant and the pellet samples were stored at -80°C and then used to perform cytokine and western blot analysis, respectively.

### 4.6. Cytokine Analysis

After quantification of total protein content with Bradford’s assay (Bio-Rad, Hercules, CA, USA), cytokine levels in supernatants of lung homogenates were analyzed using the Bio-Plex Protein Array System (Bio-plex Pro Mouse Cytokine 23-Plex Immunoassay, Bio-Rad, Hercules, CA, USA) according to the manufacturer’s instruction. The following inflammatory cytokines and chemokines were detected: IL-1α, IL-1β, L-2, IL-3, IL-4, IL-5, IL-6, IL-9, IL-10, IL-12(p40), IL-12(p70), IL-13, IL-17, Eotaxin, G-CSF, GM-CSF, IFN-γ, KC, MCP-1, MIP-1α, MIP-1β, RANTES, and TNF-α.

### 4.7. Western Blot Analysis

Pellets of lung homogenates were suspended in 500 μL of lysis buffer (25 mM MOPS pH 7.4/150 mM NaCl/1% Triton and protease inhibitors) and let to solubilize in ice for 1 h. Afterward, samples were centrifuged (20,000× *g* for 30 min at 4 °C) and supernatants were collected and stored at −80 °C). Total protein content of samples was measured by Bradford assay and a total of 20 μg of protein was used for Sodium Dodecyl Sulphate - PolyAcrylamide Gel Electrophoresis (SDS-PAGE). Prior loading, samples were mixed with SDS sample buffer containing 1,4-dithiothreitol and heat-treated (except for Fpn). Primary antibodies used for Western blots were: monoclonal anti-transferrin receptor 1 (anti-TfR) (Santa Cruz, CA, USA) (1:5,000), monoclonal anti-Fpn 31A5, generously provided by T. Arvedson (Amgen, Thousand Oaks, CA, USA) (1:10,000) [63], polyclonal anti-Ftn (Santa Cruz, CA, USA) (1:10,000) and monoclonal anti-actin (Santa Cruz, CA, USA) (1:10,000). After proper secondary antibody (Horseradish Peroxidase-conjugated), Enhanced ChemiLuminescence (ECL Prime) (GE Healthcare, Little Chalfont, UK) was used for blot development. Protein levels were normalized on β-actin by using ImageJ program.

### 4.8. Determination of Total Iron in BALF

BALF samples (100 μL) were mixed with 100 μL of 10 mM HCl and with 100 μL of a freshly prepared solution consisting of equal volumes of 1.4 M HCl and 4.5% (*w*/*v*) KMnO4 in H_2_O (iron-releasing reagent). After incubation for 2 h at 60 °C in a fume hood, samples were cooled to room temperature and 30 μL of the iron-detection reagent (6.5 mM ferrozine, 6.5 mM neocuproine, 2.5 M ammonium acetate and 1 M ascorbic acid) was added and incubated for 30 min. Thereafter, a total of 280 μL of each sample was measured at 550 nm in a 96-well plate using a microplate reader. Iron standard curve (0–300 μM of FeCl_3_) was used to determine samples’ concentrations.

### 4.9. Statistical Analysis

Statistical analysis was performed by GraphPad Prism using a two-way analysis of variance (ANOVA) with Bonferroni’s multiple comparison test for the body weight lost evaluation and Mann–Whitney U test for the other analysis. Outlier data, identified by Grubbs’ test, were excluded by the analysis. *p* < 0.05 was considered significant.

### 4.10. Ethic Statement

Animal studies were conducted according to protocols approved (3 December, 2015) by San Raffaele Scientific Institute (Milan, Italy) Institutional Animal Care and Use Committee (IACUC#733) and adhered strictly to the Italian Ministry of Health guidelines for the use and care of experimental animals.

## Figures and Tables

**Table 1:** Cytokines/chemokines levels in infected wild-type (WT) and cystic fibrosis (CF) mice treated or not treated with bovine lactoferrin (bLf).

Cytokine/chemokine (pg/mL)	WT	WT bLf	CF	CF bLf
IL-1α	95 ± 22	78±45	167 ± 93	110 ± 82
IL-1β	130 ± 32	119 ± 40	175 ± 65	124 ± 66
IL-2	ND	ND	ND	ND
IL-3	3 ± 1	3 ± 2	3 ± 1	3 ± 2
IL-4	ND	ND	ND	ND
IL-5	3 ± 1	6 ± 2	5 ± 2	5 ± 1
IL-6	9 ± 5	6 ± 4	11 ± 4	9 ± 4
IL-9	ND	ND	ND	ND
IL-10	12 ± 3	10 ± 4	17 ± 4	12 ± 4
IL-12(p40)	10 ± 6	16 ± 9	7 ± 1	9 ± 4
IL-12(p70)	69 ± 14	64 ± 18	105 ± 35	71 ± 43
IL-13	77 ± 15	83 ± 17	92 ± 13	78 ± 10
IL-17	11 ± 3	14 ± 8	5 ± 2	11 ± 6
Eotaxin	540 ± 167	417 ± 93	612 ± 149	390 ± 120
G-CSF	301 ± 216	134 ± 162	479 ± 252	283 ± 225
GM-CSF	ND	ND	ND	ND
IFN-γ	11 ± 1	12 ± 5	11 ± 2	11 ± 5
KC	90 ± 50	67 ± 29	131 ± 63	79 ± 41
RANTES	42 ± 44	63 ± 26	20 ± 21	40 ± 14
TNF-α	35 ± 14	33 ± 9	52 ± 22	38 ± 23

Cytokine/chemokine levels in murine lung homogenates after 7 treatments with aerosol administration of 50 µL sterile saline or 200 µg/50 µL bLf. IL: Interleukin; G-CSF: Granulocyte Colony-Stimulating Factor; GM-CSF: granulocyte-macrophage colony–stimulating factor; IFN: Interferon; KC: Keratinocyte Chemoattractant; TNF: Tumor Necrosis Factor. ND: Not Detected.
